# A Lock Free Approach To Parallelize The Cellular Potts Model: Application To Ductal Carcinoma In Situ

**DOI:** 10.1515/jib-2019-0070

**Published:** 2020-04-07

**Authors:** Antonio J. Tomeu, Alberto G. Salguero

**Affiliations:** University of Cadiz, Computer Science, Escuela Superior de Ingeniería, Campus of Puerto Real Puerto Real, Spain; University of Cadiz, Faculty of Engineering, Department of Computer Science, Puerto Real, Spain

**Keywords:** Cellular Automata, Cellular Potts Model, DCIS, multicore, parallel, software transactional memory, speedup

## Abstract

In the field of computational biology, in order to simulate multiscale biological systems, the Cellular Potts Model (CPM) has been used, which determines the actions that simulated cells can perform by determining a hamiltonian of energy that takes into account the influence that neighboring cells exert, under a wide range of parameters. There are some proposals in the literature that parallelize the CPM; in all cases, either lock-based techniques or other techniques that require large amounts of information to be disseminated among parallel tasks are used to preserve data coherence. In both cases, computational performance is limited. This work proposes an alternative approach for the parallelization of the model that uses transactional memory to maintain the coherence of the information. A Java implementation has been applied to the simulation of the ductal adenocarcinoma of breast *in situ* (DCIS). Times and speedups of the simulated execution of the model on the cluster of our university are analyzed. The results show a good speedup.

## 1 Introduction

**Figure 1:**
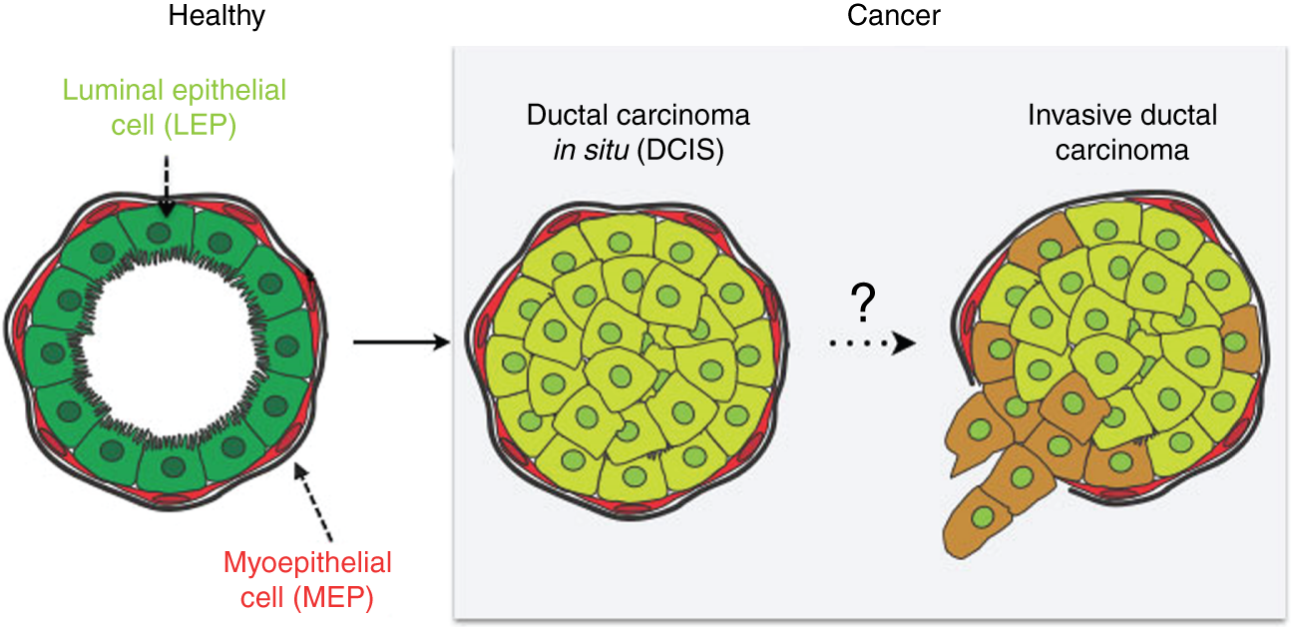
Natural history for breast adenocarcinoma ductal *in situ* (DCIS). It is observed the scheme of a mammary duct in histological cross section, and the transformation from a healthy histology to a pathological one.

The human breast is a tissue with a high architectural complexity, where the parenchyma branches out into networks of ducs formed in a cross-section(Figure 1) by an endoepithelium of luminal cells; myoepithelial cell rodeate that endoepithelium and a basement membrane subsume all the structure into the glandular stroma. The exchange of signals between the cells and their local microenvironment maintains the structure and function of the mammary epithelium. The alteration of this signalling becomes, in a first phase, a ductal carcinoma *in situ* (DCIS), and in a second, an infiltrating ductal carcinoma (Figure 1). The causes of this last phase are not yet fully known. DCIS is characterized by an abnormal and uncontrolled growth of epithelial cells that invade the duct light, but do not break the basement membrane. Sometimes, if DCIS is not treated, the infiltrating transformation occurs, which breaks the basement membrane and invades the glandular stroma. At present, it is not yet entirely clear how DCIS transforms into an infiltrating carcinoma, although the presence of mutations in the BRCA1, BRCA2, PTEN and PT53 genes is a known cause. The use of mathematical models to study the evolution of cancer has been widely used [1], [2], [3], [4]. This paper presents an alternative parallel implementation for Cellular Potts Model to those already known in the literature [5], [6], [7], [8]. Instead of using a partitioning of the data domain between parallel tasks, which forces information to be disseminated among neighboring data zones [9], [10], which entails inter-thread containment, we propose an implementation that uses a single data domain common to all tasks, along with a self-synchronized auxiliary list under software transactional memory [11] that allows this dissemination to be achieved much more efficiently. We implemented the proposed parallel model using the Java programming language and we measured time and speedup on several nodes of the cluster of processors of our university. Finally we discuss the results and propose our conclussions.

## 2 Cellular Potts Model

The Cellular Potts Model (hereafter CPM), also known as the Glazier-Graner-Hogeweg model, provides a framework [12] that allows computational simulation of complex cell behaviors at different levels of complexity [13], [14], [15], ranging from cellular organelles to organs. It also accommodates a wide range of physical processes, including intracellular reactions, diffusion into the extracellular space and modeling of the parenchymal matrix.

### 2.1 Mathematical Model

A cell in the CPM model behaves as a function of a balance of forces described by energy *H*, which is the sum of the interface energies with other cells, and the deviation of the volume of that cell from its basal value (e.g. by emitting a pseudopod). In the case of DCIS [16], the CPM model allows the simulation of tumour growth *in situ* by defining a two-dimensional grid *R*, with null boundary condition [17], [18], [19], [20]. Each node of the grid is defined by a pair of coordinates (*i*, *j*), and a *k* symbol taken in some finite alphabet of symbols Σ, representing a tag for a specific type of cell among which the model takes into account. A set of joints coordinates with the same value of *k* defines a cell. Formally, A cell can be defined as a subset S={α=(i,j,k):kis constant} of *R* which, depending on the scale of the model, may represent a cellular organelle, a cell or a tissue. The model evolves in discrete stages of time and in each one of them, a node of the grid tries to alter its location. To do this, a Monte-Carlo stochastic process [12], [14] is used, so that each node of the grid can try to change its location. This attempt is described according to the transition function described by the following equation:

(1) $$P(\alpha (i,j,k)\to \alpha (i^{\prime },j^{\prime },k^{\prime }))=\left\{ \begin{array}{ll} e^{-\Delta H/T_{m}} & \;if\;\Delta H>0\\ 1 & \;if\;\Delta H\leq 0 \end{array}\right.$$

where Δ*H* is the change in effective energy, according to the hamiltonian *H* defined below, *T_m_* is the temperature parameter and the triplet (*i*, *j*, *k*) specifies a grid node that is part of a specific cell domain *α*. The hamiltonian *H* is defined by the following equation:

(2) $$\begin{array}{rl} H & =\sum_{\left(i,j,k),(i^{\prime },j^{\prime },k^{\prime }\right)}J\tau (\alpha (i,j,k)),\tau (\alpha (i^{\prime },j^{\prime },k^{\prime }))(1-\delta_{\alpha (i^{\prime },j^{\prime },k^{\prime }),\alpha (i,j,k)})\\ & \;+\sum_{\alpha }\lambda_{V}(\tau )(V(\alpha )-V_{t}(\alpha ){)}^{2}\\ & \;+\sum_{\alpha }\lambda_{S}(\tau )(S(\alpha )-S_{t}(\alpha ){)}^{2} \end{array}$$

**Figure 2:**
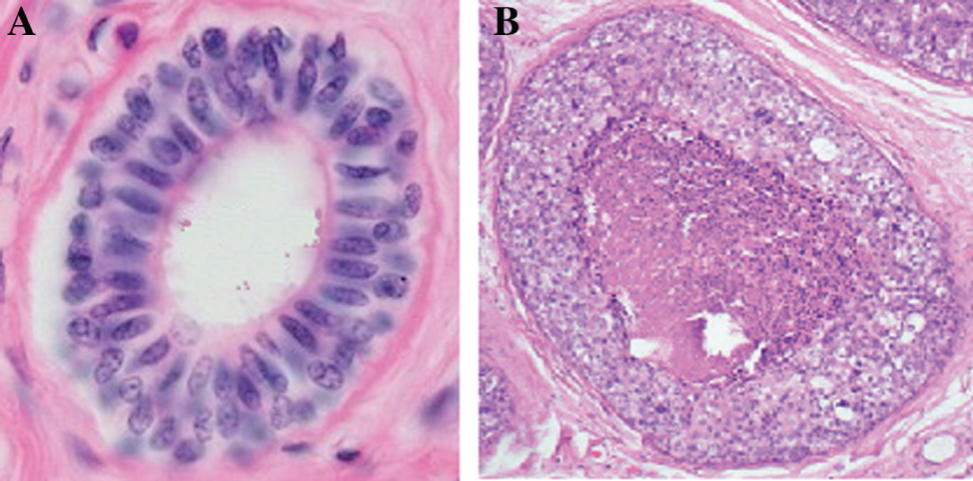
Normal histological structure (A) of a duct in the human breast, in cross section. The double layer of cells is visible. The innermost layer (elongated nucleus cells) define the luminal epithelium, and are surrounded by myoepithelial cells (rounded nucleus cells). The entire structure is surrounded by a basal membrane (in pink). In (B), a DCIS structure, where the light of the duct is invaded by cells that have undergone a neoplastic transformation.

Here, *τ* represents the type of agent. In our case, the agents will represent cells of three different classes: cells (Figure 2A1Histological images are taken from Kumar, V., Abbas, V. and Aster, J. Robbins Basic Pathology 10^th^ Edition, Elsevier. 2017.) from the extracellular matrix, luminal cells and myoepithelial cells, modeled by an alphabet $\Sigma \subset Z^{+}$ content. In the equation that defines *H*, the first term describes the energy of adhesion between a cell and its neighbors. The second term defines the volume and degree of compressibility of the agent. To do this, the difference between the volume (*V*) and the target volume (*V_t_*) is multiplied by the parameter *λ*_*V*_ which describes the rigidity of the agent. The third term of the hamiltonian models the elasticity of (*S*) and (*S_t_*) represent the surface and the target surface. *J* is an adhesion parameter.

### 2.2 Parameterization For CPM

The model, for achieving a similar structure to a cross-sectional layer of a normal breast duct (Figure 2A), consists of approximately 50 luminal cells (*L*) surrounded by an outer layer of myoepithelial cells (*M*); both layers are constructed with a circular geometry. In the simulation each cell begins as a square domain of 10 × 10 nodes with a *k*-value common to all of them. The volumetric parameters *λ*_*V*_ allow to define the elasticity of the cellular cytosol. Since in this work the interest is focused on the acceleration of the CPM model, we have chosen its values according to previously published works [5], [6], [16] that place more emphasis on the fidelity of the model to the modeled biological reality. Table 1 shows the total parameterization required in the CPM model.

**Table 1:** Parameters for CPM.

Adhesion parameters	Value
*J_L,L_*	−20
*J_L,M_*	−10
*J_M,M_*	−5
Surface parameters	Value
*S_t,L_*	−31
*S_t,M_*	1
*λ*_*S*,*L*_	31
*λ*_*S*,*M*_	1
Volume parameters	Value
*V_t,L_*	78
*V_t,M_*	5
*λ*_*V*,*L*_	78
*λ*_*V*,*M*_	10
Elasticity parameters	Value
*L_L,L_*	8
*L_L,M_*	8
*L_M,N_*	5
*λ*_*L*,*L*_	50
*λ*_*L*,*M*_	5
*λ*_*M*,*N*_	50

### 2.3 Cell Growth In CPM

In a normal duct (Figure 2A), the luminal cells remain most of the time in phase *G*_0_ of the cell cycle, without carrying out mitosis. The set of proto-oncogenes *BRCA*1, *BRCA*2, *PTEN* and *TP*53 ensure this, so that the normal structure of the duct is maintained. If a luminal cell attempts to proliferate in the light of the duct, the *PTEN* gene that induces its immediate apoptosis is activated. When the above genes accumulate mutations, the safety mechanisms described stop working [16], and the luminal cells proliferate uncontrollably in duct light forming a clone from the damaged cell, reaching DCIS (Figure 2B). In our implementation, 15% of the luminal cells were chosen to proliferate, as other authors propose for the CPM simulation of DCIS [6]. The standard implementation of the CPM model, which is the one chosen here, does not contemplate the presence of genes in the model, and we have not included them. However, it is perfectly possible to integrate this genome in the CPM model, so that Monte-Carlo processing causes mutations in them in a random way, in coherence with the rate of known mutations in epidemiological studies, and decides the behavior of a cell according to the mutational load that its genome accumulates.

### 2.4 Cellular Death In CPM

Cell death usually follows two possible routes: programmed cell death, or apoptosis, and necrosis. During the life cycle of a normal epithelial luminal cell, a cell is induced to apoptosis when it experiences mechanical stress due to an overpopulation in its immediate local environment, which transduces into the cells the signals that activate the apoptotic mechanism. In the CPM model, we have contemplated overpopulation by counting the number of cells neighboring a given cell in a radius of 2.5 cell diameter. If there are 10 cells in that vicinity, the cell has an overpopulated local environment, and will be able to enter apoptosis with a 0%–1% probability. If apotosis occurs, the cell is removed from the simulation. Necrosis depends on the gradient of available nutrients. Since during the *in situ* phase of DCIS, angiongenesis phenomena do not occur inside the duct, this gradient is high when the cells are close to the outer layer of the duct, and decreases when we move away towards the interior. This has been simulated in the model by killing by necrosis those cells located ten or more cell diameters away from the outer layer. A necrotic cell no longer interacts with its neighbors, but continues to occupy space.

### 2.5 Intercellular Adhesion In CPM

Although biologically the mechanisms that regulate adhesion are many and varied, the CPM model quantifies them using adhesion coefficients, which define the adhesion energy per unit of contact surface, and which may include aspects linked to the cultivation medium (higher energy in high nutrient presence) or necrosis (minor energy, if a neighboring cell dies). The processes of repulsion and attraction between cells are modeled by means of the surface parameters *λ*_*S*_, and again they are taken from previously published works [5], [6], [16] in the literature, respecting also the biologically contrasted hierarchy that establishes the following chain of inequalities $J_{\left(L,L\right)}<J_{\left(M,L\right)}<J_{\left(M,M\right)}$, that allow the cells to be located in a stroma of tissue with deformation capacity.

## 3 Software Transactional Memory

As is well known, the usual synchronization techniques in concurrent programming using locks to access with mutal exclusion to shared data, suffer from several problems: if we use it inappropriately or forget about it, the changes made by one thread may not be visible to the others. And at every point of the code where we must prevent a race condition, we will need to make use of them. Additionally, when we synchronize with locks, parallel threads are forced to wait, seriously affecting the degree of concurrency and the performance of our application; this, usually leads programmers to establish critical sections of very fine grain, but probably increasing the number of critical regions, and rising the probability of doing errors. By its nature, synchronization whith locks can lead to problems of liveness, since it is relatively simple leads to the application to a deadlock, as a result of a circular waiting condition between locks and poorly designed threads. Nor is it especially difficult to have threads that remain at livelock when they continually fail to achieve the lock provided by a particular synchronization point. Much has been written and researched on how to avoid all of the above [21], [22], [23], [24], and numerous are the proposed formal techniques to avoid committing the typologies of failures described. Notwithstanding the foregoing, the control of concurrency remains a complex matter, where producing a clean code is not within everyone’s reach.

**Figure 3:**
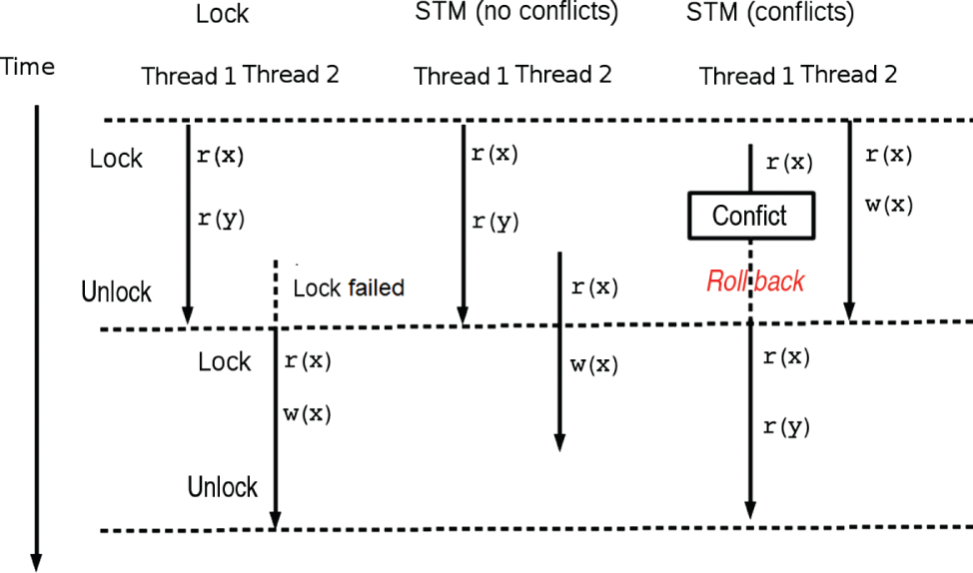
Two threads accessing shared data under three different scenarios; in the Lock scenario, it is not possible to have parallel readings due to the use of locks; in the STM scenario (no conflicts), the use of transactional memory does allow parallel readings within transactions; finally, in the STM scenario (conflicts), a task that wants to read within a transaction detects that another transaction has changed the state of the data, and performs a roll-back to obtain a consistent view of the data.

The Software Transactional Memory (STM) paradigm changes this, introducing the concept of transaction [21], which we can define as a region of code that is executed in an atomic, consistent and isolated way, without blockages. When two threads try to access the same data, the transaction handler is activated to resolve (see the Transaction Processing Algorithm below) the conflict [22], [23], without the need to use explicit locks in our code (Figure 3). When a transaction is in progress, if there is no conflict with other threads/transactions, the transaction is completed (commit) and the changes made inside it are written in memory. In another case, as soon as the transaction handler finds that another transaction has progressed beyond a point that makes the current transaction unsafe, jeopardizing the consistency of the data, it undoes the changes and tries again (roll-back). The whole process is graphically presented in Figure 3. There are multiple implementations of STM for Java [11], [24]. With STM, programmers only need to delimit in their code the access to shared data, and wrap them within a transaction. All the responsibility of maintaining the coherence of the data, and ensuring to the tasks an updated vision of world, that in the model based on locks is of the programmer, is now delegated to the transaction manager. This allows to control accesses to shared data homologable to the one available with the locks model, but in addition it optimizes the performance in the access to them, and virtually eliminates the problems of liveness. Internally, and in a totally transparent way for the programmer, the transaction manager develops a work described by the following algorithm:

Transaction Processing Algorithm Begin    (1) Private copying of shared data    (2) Make updates/change to the private copy    (3) If(shared data not modified)    (4) Then update data shared with private copy and goto(End) (*commit*)    (5) Else discard private copy and goto (1) (*roll-back*) End 

In this work, we choose an approach to STM for Java based on the functional language Clojure [11], which is interpreted by the JVM, and provides an interesting intercompatibility properties between the APIs of the two languages. In Clojure, STM uses to work a separation between the identity of an object and its state [21]. Since Clojure is a functional language, states never change; they are immutable by definition. Identity of the object is the changing thing, which is actually what is visible to the threads that use it. Since by design, in Clojure values are immutable and identities are mutable only within one transaction, there is simply no way to change the state inconsistently. Any attempt to change the identity of an object outside of a transaction is illegal, and throws an exception. Threads only have access to the identity, through the respective references, and may attempt to change that identity, within a transaction, to make the state of the object pass from the previous state to the next. Of course, if one of the thread succeeds in changing the identity, it will do so transactionally and atomically. Any other thread, attempting to change the identity in turn, will see the updated status. Since there are no blocks, the concurrency is in principle greater [11], [21], [22], [23], [24]. It is enough to have the guarantee that we offer to the threads a consistent vision of the world, and the truth is that with Clojure, it is not necessary for us to worry about doing so. The transaction handler that supports STM does it for us. Syntactically, the matter has no difficulty whatsoever. It is enough to involve in a transaction the change of identity that we want to achieve, and Clojure guarantees us that it will be done in an atomic, isolated and consistent way. When a transaction for a thread is in progress, if there is no conflict with other threads/transactions, it is completed, and the changes are written in memory (*commit*). If the transaction handler detects a conflict it stops, undoes the transaction (*roll-back*) and starts again. There is no blocking, although a little price is obviously paid for it: transactions require extra processing time [10]. The key issue here is: is this extra time greater than for the time for locks model? This work shows that for the chosen problem, and with the parallel processing algorithm with transactions developed, the answer is no.

**Figure 4:**
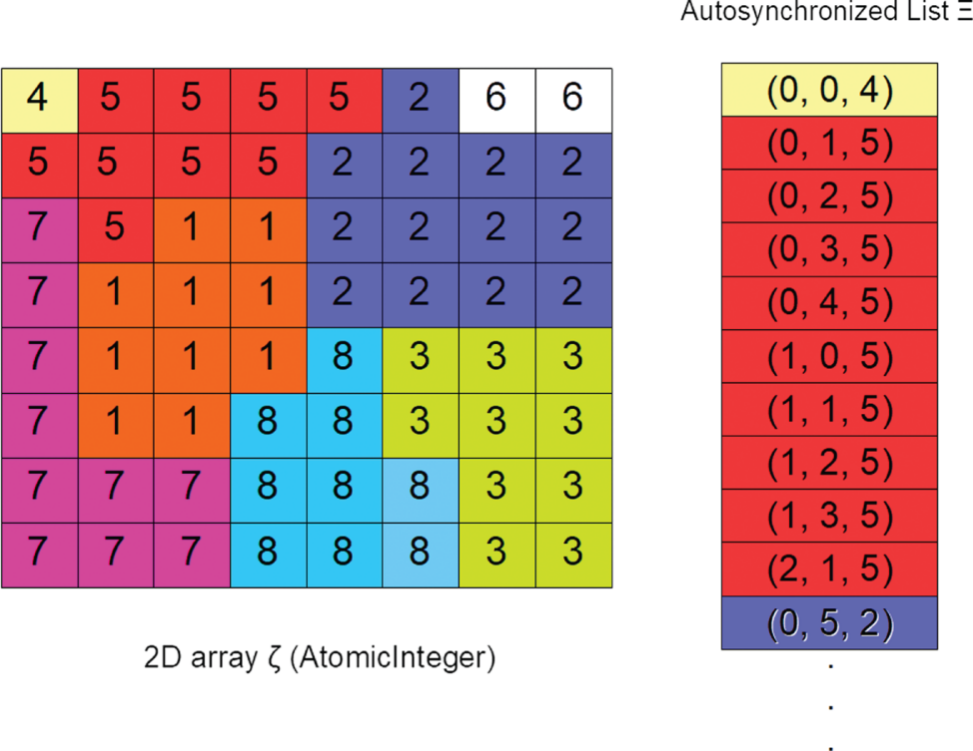
Data structures needed for parallel implementation of the Potts cell model. Several cells are illustrated (with different colors) in the grid *ζ*, and its representation in the list Ξ.

## 4 CPM Implementation With Software Transactional Memory

In order to speed up the simulation of the CPM model, we hava carried out an implementation that manages in parallel the tissue domain *ζ* in which, unlike other proposals [1], [5], [6], [7], [8], [9], the data structure is not divided between processing nodes, since this forces the propagation of information between threads that are responsible for bordering substructures, when a cell crosses the border between nodes, as a consequence of a mitosis or the extension of a pseudopod. Instead, we developed the stochastic modeling phase by dividing the number of iterations of the same into independent stochastic sub-processes, each of which uses different random generators, with different initial seeds obtained from the system clock. Data consistency is ensured using software transactional memory. The implementation for CPM that we propose in this paper uses the following data structures (Figure 4):

- A two-dimensional array *ζ* to represent the domain tissue. In *ζ* each node has an associated numeric value *k* that indicates the cell that occupies it. An *S* collection of related nodes, with the same associated numeric value, define a cell.
- A list Ξ of elements (*i*, *j*, *k*), that contains the information of those nodes of the grid *ζ* that already tried the expansion process described by equation 1. Each element of the list defines a node (*i*, *j*) of the grid *ζ*, together with the numeric value *k* that indicates which cell occupies it. If there is none, *k* = 0.

Parallel tasks/threads were coded using the Java Runnable interface, and were processed through a thread pool executor, delegating to him all the management of the tasks life cycle. Each parallel task executed the Evolve algorithm, which is illustrated below. All of them received access to the common data structures *ζ* and Ξ through specific references.

Algorithm Evolve(zeta, xi) 1. for(i=1, i<niterations/ntasks, i++){    1.1 x=random(xmax);    1.2 y=random(ymax): 2. atomic{if !((x,y) in xi) //begin transaction    2.1 cell= zeta[x][y];    2.2 xi.add((x,y,k));   }                        //end transaction 2.3 else goto line 1.1 } 3. neighbourX =getRandom(rangXMin,rangXMax); 4. neighbourY =getRandom(rangYMin,rangYMax); 5. cellNeighbour =zeta[neighbourX][neighbourY]; 6. J=getEnergyAdhesionForNeighbours(cell); 7. V=lambdaV*getVolumeForCell(cell); 8. S=lambdaS*getSurfaceCell(cell); 9. deltaH=J+V+S.> 10. if(deltaH>0)    10.1 p=Math.exp(-deltaH/params.getTemperature());    10.2 else if(deltaH<=0)p=1; 11. if(p>=random()) zeta[x][y]=cellNeighbour(); } 

**Figure 5:**
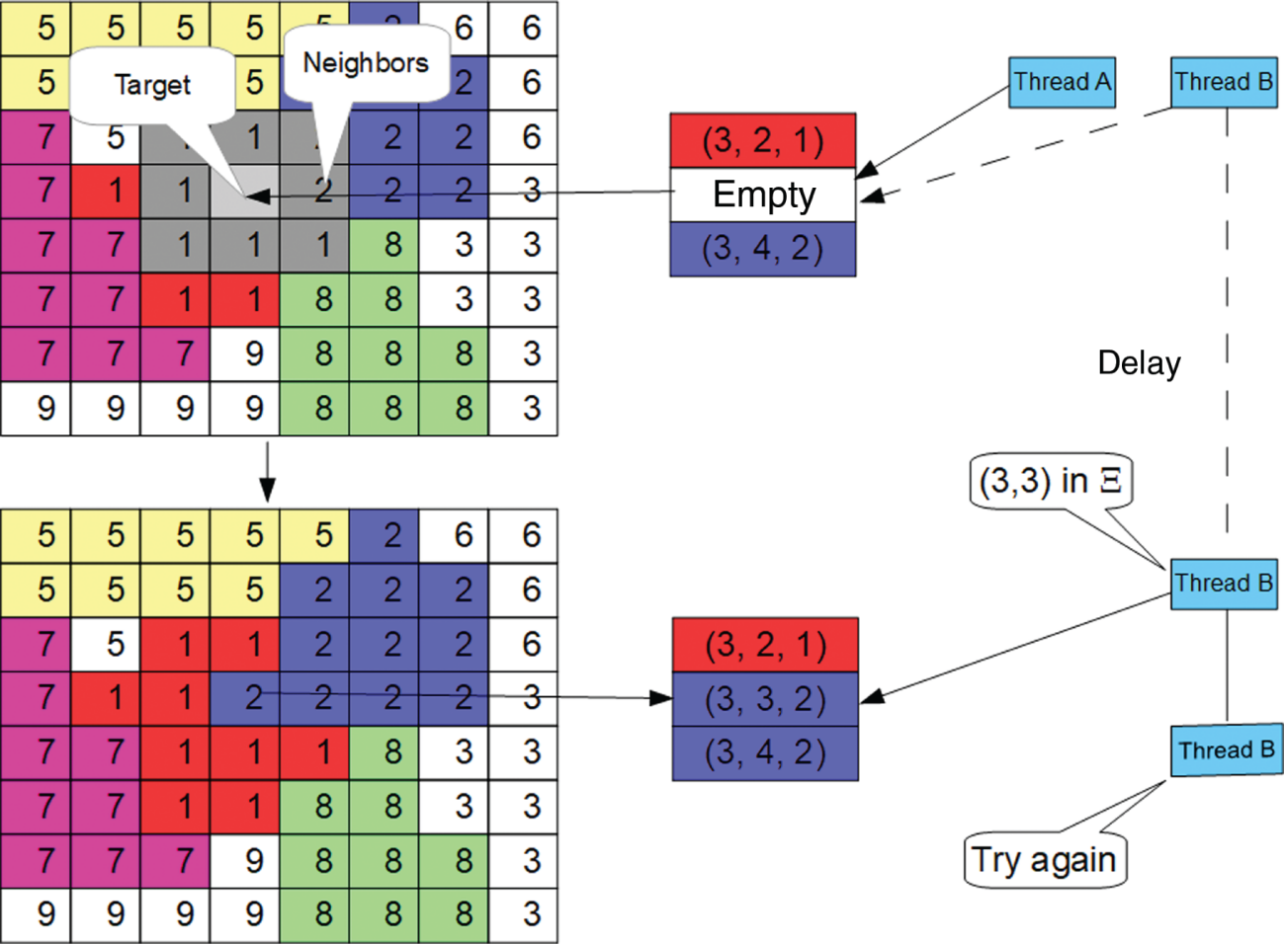
Safe parallel computing. Thread A performs a change of location of cell number 2 within a transaction, leading it to expand its cytosol. Thread B, which also attempts to do so, must undo its transaction and restart it, which is a delay.

In this algorithm, zeta and xi are the grid of cells and the list of nodes with cells processed yet; k is the value that together with the x and y coordinates defines a point of the zeta grid that is part of a cell (see the definition of cell in the section dedicated to the mathematical model); niterations is the number of steps developed by our Monte-Carlo approach, and we divide it between the number of parallel task, ntastk; xmax and ymax represent the width and height of the grid *ζ* so the random point chosen is within the grid; rangXmin, rangXMax, rangYmin, rangYMax are used to delimit a target subgrid within which the algorithm searches for a neighboring cell with which to interact; lambdaV y lambdaS are variables that represent the volume and surface parameters; getEnergyAdhesionForNeighbors, getVolumenCell and getSurfaceCell methods are used to calculate surface and volume parameters needed to determine the energy change, represented in the deltaH variable. All these variables, except zeta and chi are local and specific to each parallel task. The task that processes each thread is a direct implementation of CPM, which selects in a stochastic way (lines 1.1 and 1.2) a node of the grid (which is part of a cell) that has not yet had the opportunity to change its location. Once chosen, this information is propagated to all the other threads, inserting it in the list Ξ within a transaction. Since the list is processed from a transaction on line 2, any race condition between tasks attempting to relocate the same cell is resolved by checking the bifurcation of line 2, which is processed atomically. The task that completes its transaction (commit) updates the list and those that do not, undo the transaction (roll-back) and start it again with an updated vision of it. Since there is now a single shared data structure (the list Ξ, see Figure 3) instead of the n-1 contact zones that the usual division of the data domain between *n* tasks requires, the containment of parallel threads is reduced. From here, the different terms of the hamiltonian *H* (lines 6, 7 and 8) are determined, the probability of change of location or mitosis (conditional bifurcation of line 10) is computed and, depending on it, that change occurs or not (line 11). For a correct functioning of the parallel tasks model, it is necessary to impose some security conditions that limit its computational efficiency, but that allow guaranteeing an adequate use of the data structures between the parallel strands that process them. In particular it is necessary to use the following conditions:

- The two-dimensional array *ζ*, which has been implemented through a standard byte array in Java, must be secure against writing on specific positions of the array. This is achieved by forcing the tasks to read list Ξ beforehand, so either they get an updated view of the world, or have to wait until that vision is available. This prevents two different tasks try to process the same grid point. We have chosen this option as opposed to the one usually proposed in the literature (locks on the array or information propagation), since it is verified that this approach presents better response times than the division of the data domain between the strands, and the control of the border zones by means of locks, which also require an algorithm of information diffusion to the bordering data zones.
- The list Ξ needs to be accessed within a transaction, so that when a thread checks whether the node to be developed (line 2 of the algorithm), it does so with the updated information (Figure 5). Among the multiple options that the Java API offers, we have chosen the ArrayList class, processed within a transaction, which guarantees that all read and write operations are developed on an updated copy of the underlying array, being therefore safe against parallel threads.

Software transactional memory was supported using the extension that Clojure provides to Java for it [11], and that had already been analyzed by us in terms of performance [25].

## 5 Biological Validation For CPM Implementation

To contrast the validity of the parallel implementation of the CPM model, we have developed two different experiments that simulate two different varieties of DCIS (using the parameters described in Table 1), and compared the outputs resulting from the execution with real histological slices:

- solid variety of DCIS; in this histological subtype, myoepithelial cells of a normal duct proliferate until their cross section is completely filled with neoplastic cells.
- cribiform variety of DCIS; in this case, and with a slightly different parameterization for the model (probability of apoptosis equal to 1% and some accentuated growth directions) from the previous case, cell necrosis zones are formed that histologically appear as holes in the transversal section of the duct.

**Figure 6:**
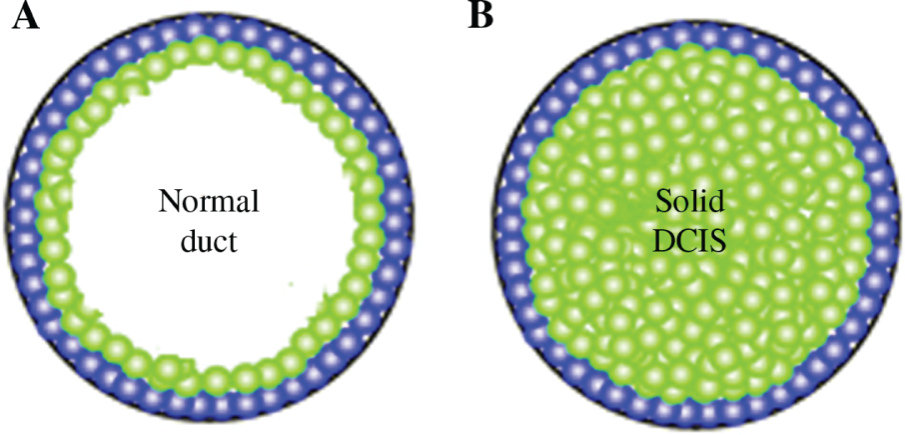
Simulation of the DCIS solid variety with the proposed parallel model. The image illustrates the initial state of the duct in cross section (A), and the final state of the duct (B) where the neoplastic cells have completely invaded its internal space.

**Figure 7:**
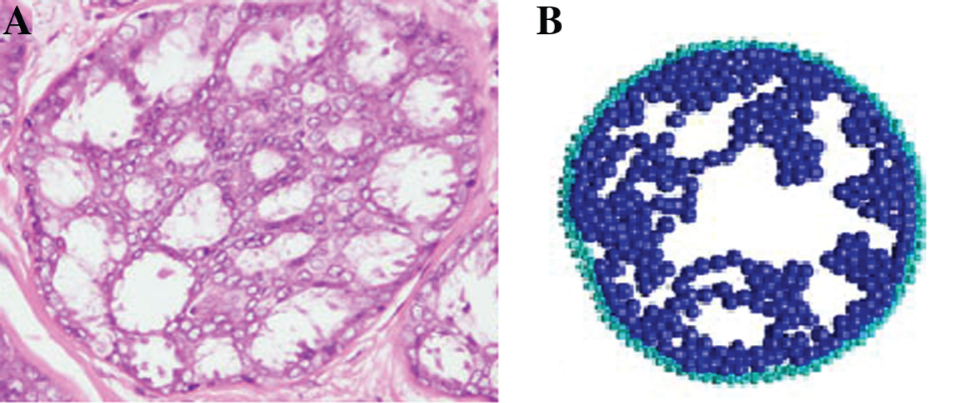
Simulation (B) of the cribiform variety of DCIS with the proposed parallel model, compared with a histological cut (A), with formation of crypts in the duct.

Figure 6 illustrates the result of the simulation from the initial normal state of a duct (a) to the final state (b) where neoplastic cells have completely invaded the luminal space, for $10^{3}$ iterations, where the number of cells in the stroma was increasing, and compatible with the gompertzian model that usually describes tumoral kinetics. Compare it with Figure 1 and Figure 2 to see the result of the simulation versus the histological cut. Similar simulations for cribiform DCIS (Figure 7A and B) were also carried out, with results consistent with their biological counterparts.

## 6 Measurements and Discussion

**Figure 8:**
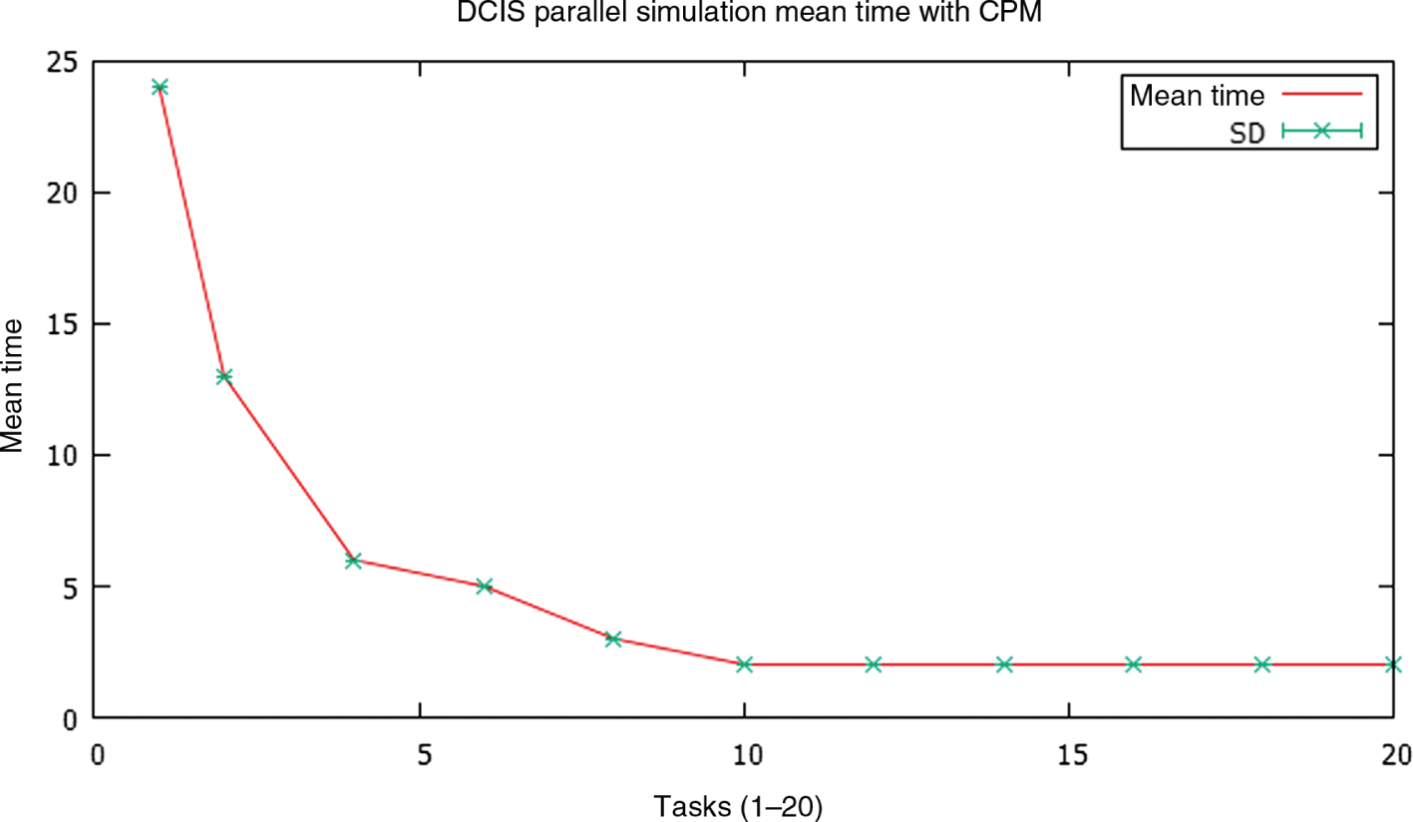
Average times for an increasing number of parallel threads, obtained by averaging the times resulting from running the simulation with the same initial conditions in eight different nodes of the cluster.

With the described implementation, tests were carried out on the cluster of processors of our university. Each node of the cluster has two processors Intel® Xeon™ E5 to 2.6 GHZ, which yield 20.8 GFLOPS together, with a common memory bank of 128 GB, and without hyperthreading. The entry node operates the HP Cluster Management Utility on Red Hat Enterprise Linux for HPC. The version of the Java development kit used was Oracle 1.8.0.151-1.b12.e17_4. The statistical treatment of the data was carried out with the R package. Runtime measurements were taken on different eight dedicated nodes of our cluster, for one execution task (sequential algorithm) and for a growing number of tasks up to 20. In all cases the initial state of the grid *ζ* is the one illustrated in Figure 6A, for a dimension of 900 × 900 nodes. Results (mean±SD times) are illustrated in Figure 8. It is observed how the proposed parallel implementation reduces the execution time as the number of parallel tasks grows, reaching a minimum mean value of 2.285 seconds for 12 tasks. Later increases in the number of tasks did not lead to additional improvements, since the delays induced by the number of transactional accesses for a higher number of tasks to the list Ξ prevented it.

**Figure 9:**
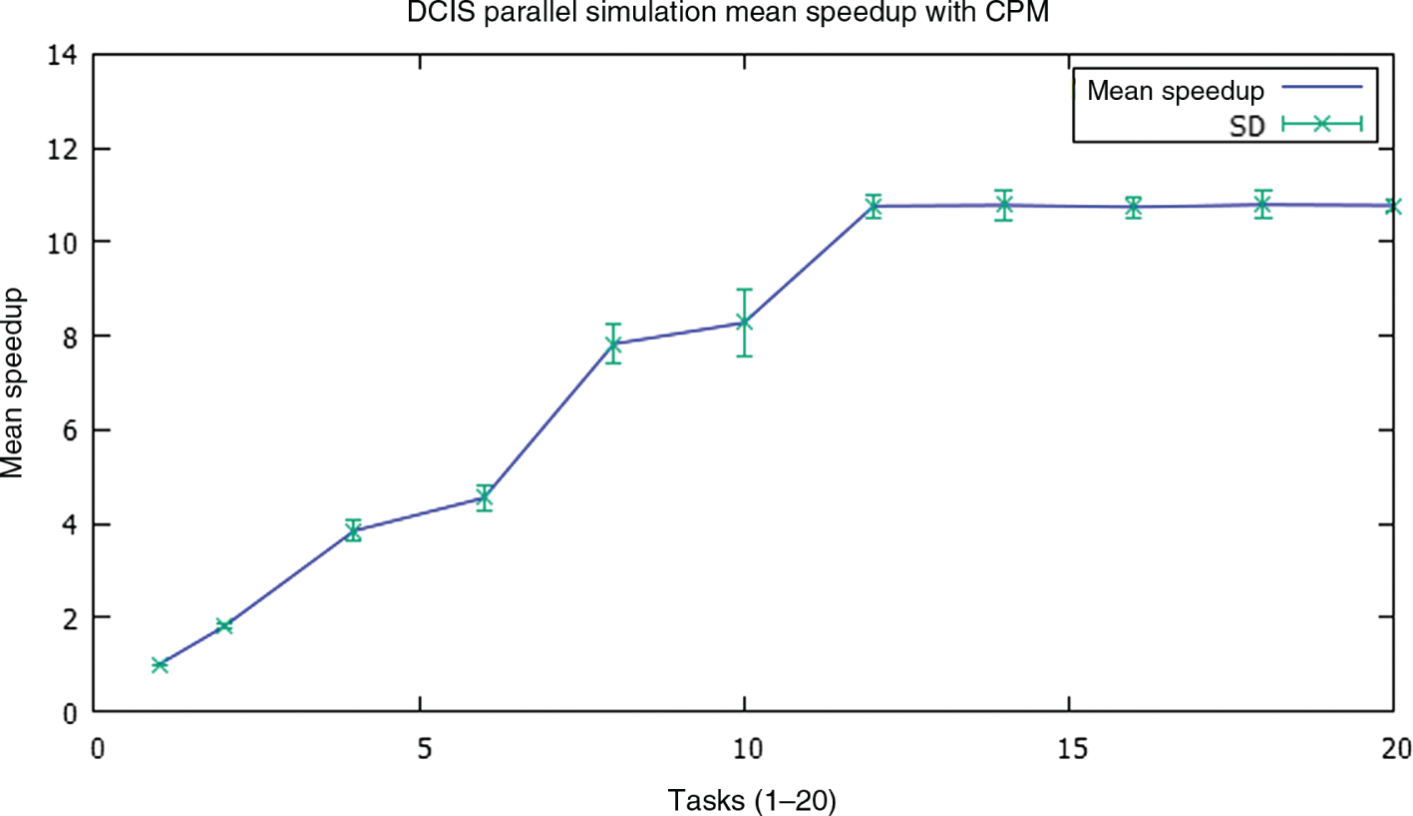
Average speedups for an increasing number of parallel threads, obtained by averaging the result of running the simulation with the same initial conditions in eight different nodes of the cluster.

Subsequently, the resulting speedups were calculated, whose mean values ± SD) are shown in Figure 9. We can see how the speedup behaves inversely to time, growing as the number of threads increases, and reaching again its optimal value for 12 tasks, with an average value of 10.76 over a theoretical maximum of 16, which is more than reasonable, and improves processing times and speedups obtained using parallelism of data on the tissue array [7], [8], or information propagation strategies between strands processing bordering tissue substructures, obtained by us [9] and by other authors [16].

**Table 2:** STM vs. locks comparison (in seconds).

Tasks	Time (STM)	Time (Locks)
2	24.48	37.98
4	13.49	22.65
6	6.37	21.36
8	3.17	11.87
10	2.87	10.40
12	2.1	10.22
14	2.15	10.34
16	2.11	10.68
18	2.12	10.67
20	2.12	10.59

Table 2 shows a comparative analysis (average time) of the STM implementation compared to another alternative implementation that has used reentrantlocks, under the same computational model. Results show a reasonable advantage of the STM control model over the lock and access under blocking model. blockage

## 7 Conclusions and Future Work

Most implementations of the CPM model suffer from the same problem: sequentiality. This seriously limits the computational biologists in the scale and duration of the simulations they can develop. There are several parallel implementations of the CPM model, which use the partitioning of the tissue domain and/or the diffusion of information between the bordering subdomains managed by parallel threads, which introduces containment in the processing of the threads. In this work we present a quick implementation of the CPM model that adds to the *ζ* grid that models the tissue domain an autosincronized list Ξ by means of transactional memory, that contains the nodes of the grid that have already been processed, and parallels the basic CPM routine between multiple parallel tasks. The list Ξ acts as an interthread synchronizing pattern, and as an access filter to the grid *ζ*, allowing it only when it is a computer node not yet processed, and always within a transaction. The tests developed by parameterizing our fast CPM model with simulation values already contrasted in the literature (Table 1) for similar DCIS, show competitive execution times (Figure 8) and even better, compared to those that other parallel implementations [5], [8] obtain, with frankly good speedup values (Figure 9). It would be possible to obtain some improvement if, instead of randomly generating cell locations and keeping the processed ones in a collection, they were taken from a previously unordered collection. We will to verify it in our future work. The rest of our future work will be oriented to the application of our proposal for rapid implementation of the CMP model to glandular tumors other than DCIS (prostate, thyroid), and to the implementation of the model on a GPU architecture. We are also interested in exploring the implementation of the model using distributed actors and agents.
